# Evolution of advanced revascularization strategies for high-risk pulmonary embolism: a physiology-guided single-center experience

**DOI:** 10.3389/fcvm.2026.1864812

**Published:** 2026-06-10

**Authors:** L. W. Greenspon, S. Whealon, J. Bonn, M. Caroline, E. Gnall

**Affiliations:** 1 Pulmonary and Critical Care, Lankenau Medical Center, Wynnewood, PA, United States; 2 Interventional Radiology, Lankenau Medical Center, Wynnewood, PA, United States; 3 Interventional Cardiology, Lankenau Medical Center, Wynnewood, PA, United States

**Keywords:** catheter directed therapy (CDT), mechanical thrombectomy, pulmonary embolism, pulmonary embolism response team (PERT), thrombolysis

## Abstract

Management of pulmonary embolism (PE) has evolved substantially over the past several decades, yet optimal integration of emerging therapies remains challenging. This Perspective describes our 4 decade, single-center experience reflecting the transition from systemic thrombolysis to catheter-directed therapies (CDT) and, more recently, large bore mechanical thrombectomy supported by interventional cardiology with ECMO back-up. Our approach has increasingly focused on physiologic assessment including right ventricular function, pulmonary artery pressures, and cardiac output in the face of the patient's age, underlying cardiopulmonary disease and presence of proximal deep vein thrombosis. This evolution has been characterized by progressive adoption of CDT, reassessment, and refinement of therapies in response to both clinical experience, emerging evidence and participation in clinical trials. The development of a pulmonary embolism response team (PERT) and more recently integration of artificial intelligence detection systems have further improved early recognition of intermediate and high-risk PE cases and coordination of care. Our experience over four decades highlights key lessons and the importance of early physiologic recognition of severity, selective use of reperfusion therapies, and the critical role of a dedicated multidisciplinary system of care.

## 1 Introduction

High-risk (massive) pulmonary embolism (PE) remains associated with substantial morbidity and mortality, largely driven by acute right ventricular (RV) failure and obstructive hemodynamic collapse (1–3). Over the past decade, catheter-based therapies and pulmonary embolism response systems have evolved rapidly; however, clinical adoption of new technologies has often preceded a full understanding of pulmonary vascular physiology, device-specific risks, and optimal patient selection. In the absence of definitive randomized trials for many advanced interventions, clinical practice has evolved incrementally as therapies emerged, demonstrated benefit, or revealed harm. Despite an expanding literature, there remains limited synthesis describing how individual centers have navigated these transitions over time, particularly in the management of high-risk PE.

Most deaths from PE occur early—often within hours to days after embolization—before effective therapy can be implemented (1, 2). While obstructive cardiogenic shock accounts for many early deaths, outcomes are also influenced by age, underlying cardiopulmonary disease, malignancy, recurrent embolization, and hemorrhagic complications of therapy (3, 4). The evolution of PE management reflects recognition that prompt anticoagulation limits clot propagation, while timely reperfusion reduces RV afterload and restores forward flow.

Recently published multi-society guidelines introduced a clinical classification system (Categories A–E) to better identify patients at risk for deterioration and mortality (5). Within this framework, Category D represents incipient cardiopulmonary failure despite preserved blood pressure, while Category E denotes overt hemodynamic instability and cardiogenic shock. Recognition of these physiologic states supports earlier consideration of reperfusion strategies, including systemic thrombolysis, catheter-based intervention, or surgical embolectomy depending on clinical context and institutional expertise.

Against this evolving framework, we describe the historical development of reperfusion strategies and systems of care at our center over four decades.

## 2 Systemic thrombolysis Era

Early randomized trials, including the Urokinase Pulmonary Embolism Trial (UPET,1974), demonstrated accelerated pulmonary reperfusion with systemic thrombolytic therapy compared with anticoagulation alone (6). Angiographic studies using Miller score demonstrated 30%–50% reduction in clot burden within 24–48 h, accompanied by significant reductions in pulmonary vascular resistance (30%–50%) and pulmonary artery pressures (7–9). Despite these early hemodynamic improvements, lung perfusion studies showed minimal differences compared with anticoagulation alone by seven days, reflecting the intrinsic fibrinolytic capacity of the pulmonary circulation (10). Mortality benefit remained not established throughout early trials.

In 1993, Goldhaber and colleagues established the widely adopted regimen of 100 mg tissue plasminogen activator (tPA) administered over two hours for high-risk PE (11). However, systemic thrombolysis carried substantial bleeding risk, including intracranial hemorrhage in approximately 2%–4% of cases (2, 3, 12).

Based on these data, thrombolysis at most centers and ours was reserved for high-risk (Category E) PE without contraindications. In elderly patients, particularly those over 75 years, reduced-dose thrombolysis was frequently employed given increased bleeding risk in this population.

In 2014, the PEITHO trial demonstrated reduced hemodynamic decompensation in intermediate–high-risk PE but increased major bleeding and intracranial hemorrhage (13). Consequently, our approach to intermediate-risk patients remained conservative.

Reduced-dose thrombolytic strategies, such as those evaluated in the MOPETT trial, suggested that partial clot reduction may be sufficient to restore pulmonary circulation and reduce RV strain (14). This concept—that complete clot removal is not required for physiologic recovery—would later become central to lower dose catheter based thrombolytic therapy and mechanical reperfusion strategies.

## 3 Recognition of Re-embolization and Inferior vena Cava filter Use

Recurrent embolization has long been recognized as a contributor to early mortality. Early studies reported re-embolization rates of approximately 3%–5% (15), while registry data from ICOPER demonstrated high mortality in unstable PE and suggested significant mortality benefit in patients who received IVC filter placement (2). From registry data in the US(National Inpatient Sample) Stein et al. suggested that both unstable and stable high risk patients that underwent surgical embolectomy had lower mortality with IVC filter placement (16).

The PREPIC-1 trial demonstrated IVC filters resulted in a reduction in early recurrent PE in patients with proximal DVT but increased long-term deep venous thrombosis (16). Proximal and free-floating DVT were associated with particularly high embolic risk (15, 17).

From approximately 1990 to 2015, temporary IVC filters were frequently used at our center in patients with intermediate–high-risk and high-risk PE when significant proximal DVT was determined by ultrasonography. Retrieval rates exceeded 90%, typically performed within 30–90 days. Following PREPIC-2, which did not demonstrate benefit of routine filter placement in anticoagulated patients (18), and with increasing reports of complications of IVC filters, filter use declined. However, in selected unstable patients—particularly those meeting Category D or E criteria—with significant proximal or mobile DVT, temporary filter placement is still being considered. In this setting, the rationale is protection against early recurrent embolization in the presence of severely compromised RV function.

## 4 Early mechanical fragmentation and rheolytic thrombectomy

Before development of PE-specific devices, mechanical clot fragmentation and rheolytic thrombectomy were explored (19). At our institution, six patients with high-risk PE and shock—including several with contraindications to thrombolysis—underwent rheolytic thrombectomy (AngioJet) with restoration of hemodynamic stability (1994–2000). However, subsequent reports identified complications including bradycardia, hypotension, renal injury, and cardiovascular collapse (20). Some effects were attributed in part to hemolysis-induced nitric oxide scavenging and transient pulmonary vasoconstriction leading to worsening pulmonary hypertension (21). Following recognition of these risks, this approach was abandoned.

## 5 Catheter-Directed thrombolysis and development of PERT (2012–2019)

In 2012, in collaboration with interventional radiology, we implemented an ultra–low-dose catheter-directed thrombolysis (CDT) protocol (0.5 mg tPA per catheter per hour for 24 h) using ultrasound-assisted delivery (USAT). [Echosonic endovascular system (EKOS)Boston Scientific,MA]. During CDT, a reduced dose of unfractionated Heparin to a PTT 40–60 s was delivered. We collected angiographic Miller scores, hemodynamics, and bleeding complications (22). Indications were standardized (Table 1) through multidisciplinary collaboration, which evolved into a formal Pulmonary Embolism Response Team (PERT). Members included Pulmonary Critical Care, Interventional Radiology, Interventional Cardiology, Emergency Medicine, Cardiothoracic surgery, Nursing and Pharmacy.

**Table 1:** Criteria for catheter-directed therapies for pulmonary embolism.

Pulmonary artery thrombus centrally located in right or left pulmonary artery with evidence of compromised distal segments and one or more of the following:
1. High-risk PE with relative or absolute contraindication for systemic thrombolysis.
2. High-risk PE with stability to be transferred to a cardiac catheterization lab within 2 h.
3. High-risk PE following systemic thrombolysis with residual hemodynamic instability.
4. High/Intermediate-risk PE defined as RV/LV ratio > 1.5 and severe RV dysfunction by transthoracic echocardiogram, positive cardiac biomarkers (troponin or BNP), signs of pulmonary hypertension, persistent tachycardia > 100 beats per minute, positive lactate > 2 mmol/L.
5. High/Intermediate-risk PE with RV/LV ratio > 1.0, positive cardiac biomarkers (troponin or BNP), and proximal deep vein thrombosis.
6. At least intermediate-risk PE with clot-in-transit in right atrium.

PE, pulmonary embolism; RV, right ventricle; LV, left ventricle; BNP, B-type natriuretic peptide.

The randomized ULTIMA trial and single arm SEATTLE II trial demonstrated improvements in RV/LV ratio and pulmonary artery pressures (23, 24) validating the efficacy of this device and procedure. Our center participated in the OPTALYSE trial (2018), which demonstrated that lower-dose regimens achieved similar efficacy with improved safety (25). The study concluded that 1 mg/catheter for 6 h was optimal. However, during the study the 2 mg/catheter for 6 h was associated with an ICH and since we had success with our 0.5 mg TPA protocol we did not change our practice. Physiologic analysis of our data identified a paradoxical rise in pulmonary artery systolic pressures after USAT with EKOS in 20% of patients (26). Whether hemolysis, distal embolization, or improved flow caused this response was not clear but reinforced the importance of pre and post hemodynamic assessment. The recent Hi-PIETHO study has confirmed the safety and efficiency of low dose thrombolytic CDT in intermediate high-risk patients (27).

## 6 Mechanical thrombectomy Era (2019–present)

In 2019, our program transitioned fully to large-bore mechanical thrombectomy (FlowTriever, Inari Medical, Irvine, CA) with support from interventional cardiology. All procedures were performed in the cardiac catheterization laboratory with invasive hemodynamic monitoring and availability of VA-ECMO. Procedures were performed on anticoagulation with Low molecular weight heparin. Clinical studies including FLARE, and FLASH have demonstrated significant improvement in RV function and low mortality in intermediate high-risk and in high-risk patients (28, 29). We participated in the FLAME trial (30) which demonstrated a 1.9% mortality in high-risk PE patients stable enough to be mobilized and undergo mechanical thrombectomy. Our experience confirmed that complete clot removal is not required. Removal of sufficient proximal thrombus to restore cardiac output frequently leads to rapid hemodynamic recovery. Recent clinical trials have supported the benefits and safety of mechanical thrombectomy for intermediate high-risk patients (Class D) (31, 32).

## 7 Identification of patients at risk for decompensation

Although intermediate–high-risk (Category D) patients are normotensive, approximately 5%–10% may deteriorate (33–35). Markers of impending decompensation include: elevated shock index, persistent tachycardia, elevated lactate, worsening hypoxemia, and a reduced cardiac index.

These findings support the concept of normotensive shock, in which circulatory failure exists despite preserved blood pressure and that it may be an additional clinical state at higher risk for decompensation. Society guidelines now support consideration of revascularization for these patients. Our current research has identified that end-tidal CO2 below 20 mm Hg on presentation may be another quick noninvasive signal to detect a higher severity PE (36).

Very early, Echocardiography became central not only for assessment of RV dysfunction but for detection of clot-in-transit (CIT). CIT occurs in approximately 3%–5% of cases and represents a highly unstable phenotype with immediate embolic potential (37). In the presence of RV dysfunction (Categories C,D/E), detection of CIT prompted urgent consideration of definitive revascularization therapy.

## 8 Systems of care

The development of a multidisciplinary PERT program enabled rapid triage and coordinated care (38, 39). More recently, AI-based detection systems have enabled early identification and activation. We are currently using Viz.ai (SF, CA). PERT team members meet quarterly to discuss cases and processes of care.

## 9 Surgical embolectomy

Surgical embolectomy remains an important option, particularly when interventional capability is unavailable, when thrombolysis is contraindicated, and when ECMO (40) support is required for patient stabilization. The number of surgical embolectomies has been significantly reduced since the mechanical CDT program started.

## 10 Acute-on-Chronic PE and CTEPH

Acute PE may occur on a background of chronic thromboembolic disease. As well, approximately 2%–4% of patients may develop CTEPH (41) after an acute event. We follow our acute PE patients in the outpatient clinic for the first 6 months and have found an exceeding low rate of CTEPH consistent with the 2% range. We are currently recruiting patients for the PETRACT NIH sponsored study (42) which will shed more light on the role of CDT for intermediate risk (Class C,D)PE in preventing this long-term complication.

## 11 Discussion

**Figure 1:**
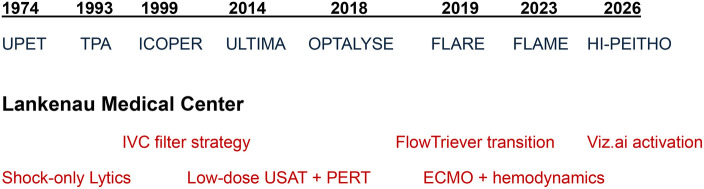
Evolution of PE revascularization: trials and institutional practice.

This single-center experience reflects the evolution of pulmonary embolism management from systemic thrombolysis to targeted mechanical reperfusion guided by physiologic principles, multidisciplinary coordination, and increasingly rapid recognition. A timeline can be seen in Figure 1, highlighting important clinical trials and our institutional development.

Several key lessons emerge:

1. Patient vulnerability modifies outcomes. Age, malignancy, and underlying cardiopulmonary disease significantly influence mortality independent of clot burden.
2. Early physiologic recognition is essential. Blood pressure alone is an insensitive marker of circulatory compromise.
3. A subset of intermediate–high-risk patients will deteriorate. Approximately 5%–10% progress despite initial stability.
4. Normotensive shock represents an important clinical phenotype. Impaired cardiac output may exist despite preserved systemic pressure and should prompt consideration of escalation and revascularization.
5. Partial clot reduction is sufficient for recovery. Restoration of forward flow—not complete clot removal—is the primary physiologic goal.
6. Re-embolization remains clinically relevant in selected patients. Particularly in the setting of marginal RV reserve and proximal DVT.
7. Thrombolytic therapy carries meaningful hemorrhagic risk. Reinforcing selective use for high-risk PE and dose minimization strategies for the elderly and with CDT strategies.
8. Multidisciplinary systems of care improve outcomes. PERT models enable rapid triage, coordinated decision-making, and continuous quality improvement.
9. Procedural programs require experience. Centers must maintain sufficient case volume to effectively manage unstable patients when they present.
10. ECMO provides life-saving support in refractory shock. Particularly when pulmonary arterial obstruction limits effective forward flow.
11. Mechanical thrombectomy enables rapid physiologic recovery. With immediate hemodynamic assessment, revascularization, and integration with ECMO support when needed.

Overall, the structured, team-based approach developed through our PERT program has enabled consistent, organized care that continues to evolve with emerging evidence and technology.

## Data Availability

The original contributions presented in the study are included in the article/Supplementary Material, further inquiries can be directed to the corresponding author.
